# Medical Ethics in the Sixteenth Century

**Published:** 1900-07-14

**Authors:** 


### MEDICAL ETHICS IN THE SIXTEENTH CENTURY,

BEING "Certain Precepts meet for young Students in Chirurgerie," appended as a final chapter to a Treatise on Gunshot Wounds, by William Clowes, published in 1591 :—

"I read that Aristotle, the wise and grave philosopher,
Wrote an Epistle unto noble King Alexander:
Saying, Choose your Servitors by their good and comely face,
For such men are most meete, to be about your Grace.
Of the same opinion, the best learned sure are still,
That the countenance doth bewray the manners good or ill.
Therefore, saith Guido, you shall in no way choose,
Any such deformed person, Chirurgery to use.
But one that is ingenious, and apt for to devise,
New remedies for new griefes, as daily they doe rise.
With a cunning, speedy, handsome handling of the griefe,
By the third part of Physick, procuring safe reliefe.
The things that a good Surgeon, ought chiefly to know,
Are naturall, not naturall, against Nature also.
Yet they that have learning, without practice in the Art,
Doe oft more hurt, than helpe, unto the grieved part.
So practice without learning, yee ought not to admit,
These two may not be separate, that are so duly knit.
There must be a dexterity, and a finenesse in working,
A quick remembrance, and a ready understanding.
He must be circumspect, and seeke to avoid all slaunder,
Not too covetous for money, but a reasonable demaunder.
Being good unto the poore, let the rich pay therefore,
So God will bless thy doings, and thou shalt have the more.
He must also be honest, and in living very upright,
To serve the Lord our God must be his whole delight,
Avoiding of all drunkennesse, and vile riot to detest,
Lest he grow fit for nothing, but Bacchus belly feast.
His fingers must be small, and his hands without quaking,
Steadfast to hold, without trembling or shaking.
Who worketh upon man's body, being unskilful of the same,
Is fitter for the stable, than to cure the sicke or lame.
The patient's lawful secrets, you ought for to conceale,
It is not for a Surgeon's credit, things secret to reveale.
Likewise the patient ought to suffer, and duly to observe,
The precepts of his Surgeon, fro the which he may not swerve.
Having good trust in him, and a sure hope and confidence,
And, touching all the cure, yeelding due obedience.
A Surgeon should not take in hand any cure or maladie,
The which is past all helpe, or hope of his recoverie;
And he that setteth a day, when his patient shall be cured,
Is but a childish Surgeon, you may well be assured.
Hippocrates in his Aphorisme, as Galen writeth sure,
Said, foure things are needful to every kind of cure.
The first, saith he, to God belongeth the chiefest part,
The second to the Surgeon, who doth apply the art.
The third unto the Medicine, that is Dame Nature's friend,
The fourth unto the patient, with whom I here will end.
How then may a Surgeon appoint a time, day, or houre,
When three parts of the cure are quite without his powre.
All these things should be observed by Surgeons as their vowes,
I wish we all could follow this, finis, William Clowes."

